# Estimated Burden of Metabolic Dysfunction–Associated Steatotic Liver Disease in US Adults, 2020 to 2050

**DOI:** 10.1001/jamanetworkopen.2024.54707

**Published:** 2025-01-17

**Authors:** Phuc Le, Moosa Tatar, Srinivasan Dasarathy, Naim Alkhouri, William H. Herman, Glen B. Taksler, Abhishek Deshpande, Wen Ye, Olajide A. Adekunle, Arthur McCullough, Michael B. Rothberg

**Affiliations:** 1 Center for Value-Based Care Research, Cleveland Clinic, Cleveland, Ohio; 2 University of Houston School of Pharmacy, Houston, Texas; 3 Department of Inflammation and Immunity, Lerner Research Institute, Cleveland, Ohio; 4 Department of Gastroenterology, Hepatology, and Nutrition, Digestive Disease and Surgery Institute, Cleveland Clinic, Cleveland, Ohio; 5 Department of Hepatology, Arizona Liver Health, Tucson; 6 University of Michigan School of Public Health, Ann Arbor; 7 University of Michigan School of Medicine, Ann Arbor

## Abstract

**Importance:**

Metabolic dysfunction–associated steatotic liver disease (MASLD) is the most common cause of chronic liver disease and is projected to become the leading indication for liver transplant (LT) in the US. Understanding its clinical burden can help to identify opportunities for prevention and treatment.

**Objective:**

To project the burden of MASLD in US adults from 2020 to 2050.

**Design, Setting, and Participants:**

This decision analytical modeling study used an agent-based state transition model that simulates the natural history of MASLD progression among adults 18 years of age or older. Primary data sources for model inputs were the published literature.

**Exposure:**

Natural history of MASLD.

**Main Outcomes and Measures:**

Cases of MASLD, metabolic dysfunction–associated steatohepatitis (MASH), fibrosis, hepatocellular carcinoma (HCC), LT, and liver-related death.

**Results:**

The model simulated 2 821 624 individuals (mean age. 35.8 years; 50.9% female). The model predicted a steady increase in the prevalence of MASLD from 33.7% (86.3 million people) in 2020 to 41.4% (121.9 million people) by 2050. Cases of MASH would increase from 14.9 million (5.8% of US adults) in 2020 to 23.2 million (7.9% of US adults) by 2050. The number of cases of MASH and clinically significant fibrosis (ie, F≥F2, centrilobular and periportal fibrosis or more severe disease) were estimated to increase from 6.7 million to 11.7 million. By 2046 to 2050, MASLD would cause 22 440 new cases of HCC and 6720 new cases of LT per year compared with 11 483 new cases of HCC and 1717 new cases of LT in 2020 to 2025. Liver-related mortality was estimated to increase from 30 500 deaths (1.0% of all-cause deaths in adults) in 2020 to 95 300 deaths (2.4%) in 2050.

**Conclusions and Relevance:**

In this decision analytical modeling study, the model forecast a substantial increase in clinical burden of MASLD over the next 3 decades in the absence of effective treatments. These results suggest that health systems should plan for large increases in the number of HCC cases and in the need for LT.

## Key Points

**Question:**

What is the projected burden of metabolic dysfunction–associated steatotic liver disease (MASLD) in US adults in the next 30 years?

**Findings:**

Using a microsimulation approach that included 2 821 624 individuals at the start, this decision analytical modeling study estimated that the prevalence of MASLD would increase from 33.7% in 2020 to 41.4% in 2050, which translated to approximately 122 million US adults having MASLD in 2050. After 30 years, prevalent cases of decompensated cirrhosis would more than triple, while incident cases of liver cancer would almost double and liver transplant would almost quadruple.

**Meaning:**

The findings of this study suggest that in the absence of effective treatments, health systems should plan for large increases in liver cancer and transplant.

## Teaser

This decision analytical modeling study estimates the burden of metabolic dysfunction–associated steatotic liver disease among US adults from 2020 to 2050.

## Introduction

The prevalence of metabolic dysfunction–associated steatotic liver disease (MASLD), formerly known as nonalcoholic fatty liver disease (NAFLD), has increased worldwide.^1,2^ By 2019, 38% of adults in North America or Australia had MASLD, and 5% had metabolic dysfunction–associated steatohepatitis (MASH), formerly known as nonalcoholic steatohepatitis (NASH).^1,3^ People with MASLD or MASH are at increased risk of adverse liver-related health outcomes,^4,5,6,7^ and MASH is projected to soon become the leading indication for liver transplant (LT) in the US.^8,9,10,11^ The new nomenclature of MASLD was adopted to replace NAFLD in 2023. While their definitions differ, approximately 99% of patients with NAFLD would be classified as having MASLD.^12,13^

Weight loss and exercise, pioglitazone, and glucagon-like peptide 1 receptor agonists can improve MASH and potentially improve fibrosis.^14,15,16,17^ Resmetirom was approved for treatment of MASH and fibrosis stage F2 and F3 (based on the Nonalcoholic Steatohepatitis Clinical Research Network scoring system, F0 indicates no fibrosis; F1, centrilobular pericellular fibrosis; F2, centrilobular and periportal fibrosis; F3, bridging fibrosis; and F4, cirrhosis), while other molecules are being tested.^18,19,20,21^ Understanding the clinical burden of MASLD, especially the number of patients eligible for pharmacologic treatment (ie, patients with MASH and fibrosis stage F≥F2), could enable health systems and pharmaceutical companies to prepare to meet imminent demand, but estimates vary widely, posing planning challenges. Estimates of MASLD prevalence circa 2016 ranged from 64 million to 83 million cases, while estimates of MASH prevalence ranged from 5.5 million^22^ to 16.5 million^23^ cases in the US. By 2030, cases of MASLD could increase to 101 million and MASH to 27 million^23^ or to less than half^24^ of that. These studies all used Markov cohort models, which assume that cohorts are homogeneous and individuals move between health states with fixed transition probabilities and fail to capture the heterogeneity in disease progression within populations. Using a microsimulation approach and the most up-to-date data on disease progression, we aimed to estimate the disease burden of MASLD, including cases of MASLD, MASH, hepatocellular carcinoma (HCC), and LT and liver-related deaths from 2020 to 2050 among US adults.

## Methods

### Study Design

This decision analytical modeling study was approved by the Cleveland Clinic Institutional Review Board. The results were reported following the Strengthening the Reporting of Empirical Simulation Studies (STRESS) guidelines.^25^

**Figure 1.**
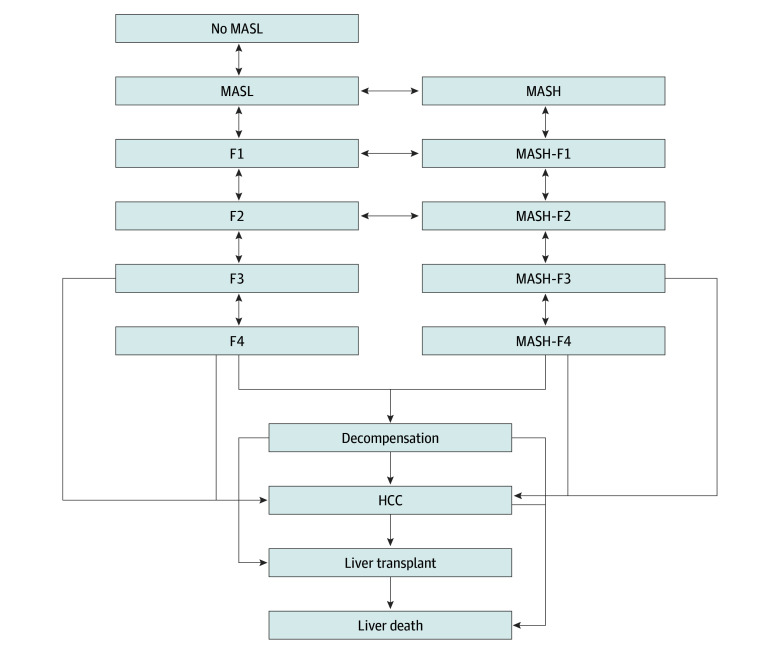
Model Diagram of the Progression of Metabolic Dysfunction–Associated Steatotic Liver Disease (MASLD) In each cycle, people can die from other causes of death from any of these health states. F0 to F4 represent fibrosis stage 0 (no fibrosis) to 4 (cirrhosis); HCC, hepatocellular carcinoma; MASH, metabolic dysfunction–associated steatohepatitis, MASL, metabolic dysfunction–associated steatotic liver.

We developed an agent-based state transition model with a yearly cycle and lifetime time horizon. An agent-based or microsimulation approach was used to capture heterogeneity within the population, account for individual-level variation, and track the impact of that variation on individual outcomes, leading to a more accurate projection within a population.^26^ The model has 2 components. The first represents a hypothetical population with age and sex distribution reflecting the US population in 2000 simulated until 2050. At the model start, we simulated 2 821 624 individuals with a mean age of 35.8 years and 49.1% male. Every year, we incorporated births and immigrants based on US Census Bureau data and mortality based on life tables.^27^ The second component tracks the natural history of MASLD in adults (aged ≥18 years). The following health states were modeled: no steatosis, simple steatosis or metabolic dysfunction–associated steatotic liver (MASL), MASH, fibrosis with or without MASH, cirrhosis, decompensated cirrhosis (DC), HCC, LT, and liver-related death (Figure 1). Fibrosis was categorized into 5 stages, from F0 (no fibrosis) to F4 (cirrhosis). Individuals occupy only 1 health state at a time. At the end of each cycle, an individual may remain in the same state or move to a more or a less severe state with predefined probabilities. We modeled MASL and MASH as parallel conditions. Patients can transition between steatosis and MASH among fibrosis stages F0 to F2. Liver-related mortality included deaths from HCC, DC, and LT.

### Model Inputs, Data Sources, and Assumptions

For each input of the natural history model component, we conducted a literature review of US-based studies to reflect MASLD epidemiology in the adult population. For unknown parameters, we used calibration to estimate their values. The Table presents key parameters and sources,^28,29,30,31,32,33,34,35,36,37,38,39^ and eTable 1 in Supplement 1 lists model assumptions.

**Table.** Annual Transition Probabilities Per Person Among Model Health States

Input	Base-case value	Range	Source
Incidence of MASLD	Varied by age and year	NA	Allen et al,^28^ 2018 and calibration
MASL to no steatosis	Half of incidence		Le et al,^29^ 2023 and assumption
No MASH to MASH	0.0060	NA	Calibration
MASH to no MASH	0.0130	NA	Calibration
Among MASL			
MASL to F1	0.0662	0.0556-0.0766	Le et al,^29^ 2023
F1 to MASL	0.0458	0.0371-0.0543	
F1 to F2	0.0741	0.0634-0.0848	
F2 to F1	0.0778	0.0638-0.0916	
F2 to F3	0.0690	0.0557-0.0821	
F3 to F2	0.0948	0.0806-0.1088	
F3 to F4	0.0605	0.0489-0.0720	
F4 to F3	0.0768	0.0557-0.0974	
Among MASH			
MASH to MASH F1	0.0984	0.0644-0.1312	Le et al,^29^ 2023
MASH F1 to MASH	0.0428	0.0284-0.0569	
MASH F1 to MASH F2	0.0981	0.0772-0.1186	
MASH F2 to MASH F1	0.0638	0.0452-0.0821	
MASH F2 to MASH F3	0.0907	0.0689-0.1120	
MASH F3 to MASH F2	0.1269	0.1046-0.1485	
MASH F3 to MASH F4	0.0779	0.0599-0.0957	
MASH F4 to MASH F3	0.0766	0.0519-0.1007	
F4 or MASH F4 to DC	0.0268	0.0250-0.0286	Bhala et al,^30^ 2011 Nyberg et al,^31^ 2021 Sanyal et al,^32^ 2019
F3 or MASH F3 to HCC	0.0011	0.0004-0.0018	Sanyal et al,^33^ 2021
F4 or MASH F4 to HCC	0.0022	0.0016-0.0027	Bhala et al,^30^ 2011 Nyberg et al,^31^ 2021 Sanyal et al,^33^ 2021
DC to HCC	0.0011	0.0008-0.0027	Assumption
DC or HCC to liver transplant	0.0062	NA	Calibration
Mortality			
DC	0.0734	0.0689-0.0779	Nyberg et al,^31^ 2021 Vilar-Gomez et al,^34^ 2018 Sanyal et al,^35^ 2006
HCC	Varied by years since diagnosis	NA	SEER program^36^
Liver transplant	Varied by age at transplant and years since transplant	NA	UNOS data^37^
Other causes of death	US life tables	NA	US life tables^38^
HR for MASLD vs no	1.15	NA	Younossi et al,^39^ 2024

Abbreviations: DC, decompensated cirrhosis; F1 to F4, fibrosis stages 1 to 4, with higher numbers indicating great severity; HCC, hepatocellular carcinoma; HR, hazard ratio; MASH, metabolic dysfunction–associated steatohepatitis; MASL, metabolic dysfunction–associated steatotic liver; MASLD, metabolic dysfunction–associated steatotic liver disease; NA, not applicable; SEER, Surveillance Epidemiology and End Results Program; UNOS, United Network for Organ Sharing.

#### Incidence and Prevalence of MASLD

The estimates of MASLD incidence vary depending on the diagnostic methods and study population. All 16 studies included in a recent meta-analysis of MASLD incidence were conducted outside the US.^2^ For our study, we derived incidence from a longitudinal community-based study in Olmsted County, Minnesota.^28^ To project future incidence, we fit 3 linear regression models corresponding to 3 different age groups with age-specific incidence as a dependent variable and the year as an independent variable (eTables 2-4 in Supplement 1). We then estimated age-specific incidence rates, using calibration with prevalence from 2001 through 2018 as targets. In the base case, we assumed MASLD incidence would increase until 2030 before stabilizing and varied this assumption in sensitivity analyses.

To populate the model with the prevalence of MASLD in 2000, the starting point of our simulation, we estimated the prevalence of MASLD in 2018 using data from the 2017 through 2018 National Health and Nutrition Examination Survey (NHANES)^40^ and conducted back calculations to determine the prevalence in 2000 (eMethods, eTables 5 and 6, and eFigure 1 in Supplement 1).

#### Progression of MASLD and Proportion of MASH

To estimate transition probabilities among MASLD-related health states, we conducted a meta-analysis of paired biopsy studies and reported the incidence of progression and regression by fibrosis stage (F0-F4) and MASH status.^29^ We estimated the rate of resolving simple steatosis to be half the incidence, based on our meta-analysis. Because published studies did not report development and resolution of MASH by fibrosis stage, we initially applied uniform rates from the meta-analysis for all fibrosis stages and then calibrated them using the proportion of MASH among MASLD from 2001 through 2018 as targets. Transition from cirrhosis (F4) to DC was independent of MASH status.^30,31,32,33,41^

In a recent meta-analysis, the proportion of MASH was found to be 60.6% (95% CI, 49.6%-70.7%) among patients with MASLD with an indication for liver biopsy and 29.9% (95% CI, 22.7%-38.1%) among patients without.^42^ Up to 5% of US adults may have MASH,^23^ but there were no data on the proportion of MASH in year 2000 or trends over time. We developed a MASH prediction model using a subset of the NASH Clinical Research Network (CRN) databases 1 through 3. Subsequently, we employed the prediction model and NHANES data to estimate the MASH proportion among patients with MASLD in 2018 and assess its temporal change. Additionally, we performed back calculations to determine the MASH proportion in year 2000 (eAppendix 1 and eTables 7-9 in Supplement 1).

#### HCC and LT

In our model, we allowed transition from F3 or F4 to HCC based on pooled longitudinal studies.^30,31,33,34^ Although people with DC can develop HCC, it is difficult to determine which occurs first. Incidence of HCC among people with DC appears low.^31,33^ We assumed the transition from DC to HCC was half that from F4 to HCC in the base-case analysis and varied it in sensitivity analyses. Finally, we assigned the same LT rates for HCC and DC, then calibrated them based on United Network for Organ Sharing (UNOS) data.^37^

#### Mortality

Age- and sex-specific background mortality rates were based on US life tables.^38^ Patients with MASLD had an increased all-cause mortality.^39^ Mortality from HCC was based on data from the Surveillance, Epidemiology, and End Results (SEER) 9 areas for 1975 through 2015.^36^ Mortality from DC was pooled from longitudinal studies.^31,34,35^ Mortality associated with LT was based on UNOS data by age at transplant and year after transplant for all transplants performed from 2008 through 2015.^37^ Deaths from cardiovascular disease and other competing risks were accounted for in the background mortality.

#### Model Calibration and Validation

We validated model outputs against several targets (eAppendix 1 in Supplement 1). We first compared the model’s population projection to the actual US population from 2001 through 2020. Second, we compared the predicted prevalence of MASLD and the MASH proportion from 2001 through 2018 against NHANES. Third, we compared the model prediction of the number of HCC cases and LTs from 2001 through 2020 against reported data from SEER and UNOS. Finally, we simulated a hypothetical patient cohort with characteristics similar to patients enrolled in the NASH CRN database and compared model-predicted vs observed outcomes.^33^

### Statistical Analysis

Model outcomes included annual estimates of the number of MASLD cases, proportion of MASH, distribution of cases by fibrosis stage and MASH status, and number of HCC cases, LTs, and liver-related deaths. The model was constructed in AnyLogic, version 8.8.4 (AnyLogic Company). Model run time was 40 minutes per iteration.

We conducted different sensitivity analyses to assess the impact of model inputs on outcomes. We explored the impact of MASLD incidence in 2 scenarios: (1) incidence remained stable after 2014, the last follow-up year available^28^ (best case), and (2) incidence kept rising after 2030 (worst case). We also predicted outcomes using 95% CIs of the rates of increase in the prevalence of MASLD and the proportion of MASH over time. Finally, we conducted one-way analyses varying the disease transition probabilities within their 95% CIs while keeping the rates of increase in MASLD prevalence and MASH proportion at their base-case values (eAppendix 1 in Supplement 1).

## Results

### Model Validation and Calibration

The model simulated 2 821 624 individuals (mean age, 35.8 years; 50.9% female and 49.1% male). Our model accurately replicated the growth of the US population from 2000 to 2020, with all yearly estimates within 0.5% of observed data (eTable 10 in Supplement 1). In addition, the overall prevalence of MASLD among US adults predicted by the model from 2001 through 2018 closely matched estimates from NHANES 2001 through 2018, as did the age-specific prevalence in 2018 and the proportion with MASH (eTable 6 and eFigures 2 and 3 in Supplement 1). The number of HCC cases predicted by the model reasonably matched the number of MASLD-related HCC cases reported from SEER, and LT projections matched UNOS data (eFigures 4 and 5 in Supplement 1). Finally, the survival curve estimated from our model matched observed survival in the NASH CRN database (eFigure 6 in Supplement 1).

### Prevalence of MASLD and MASH

**Figure 2.**
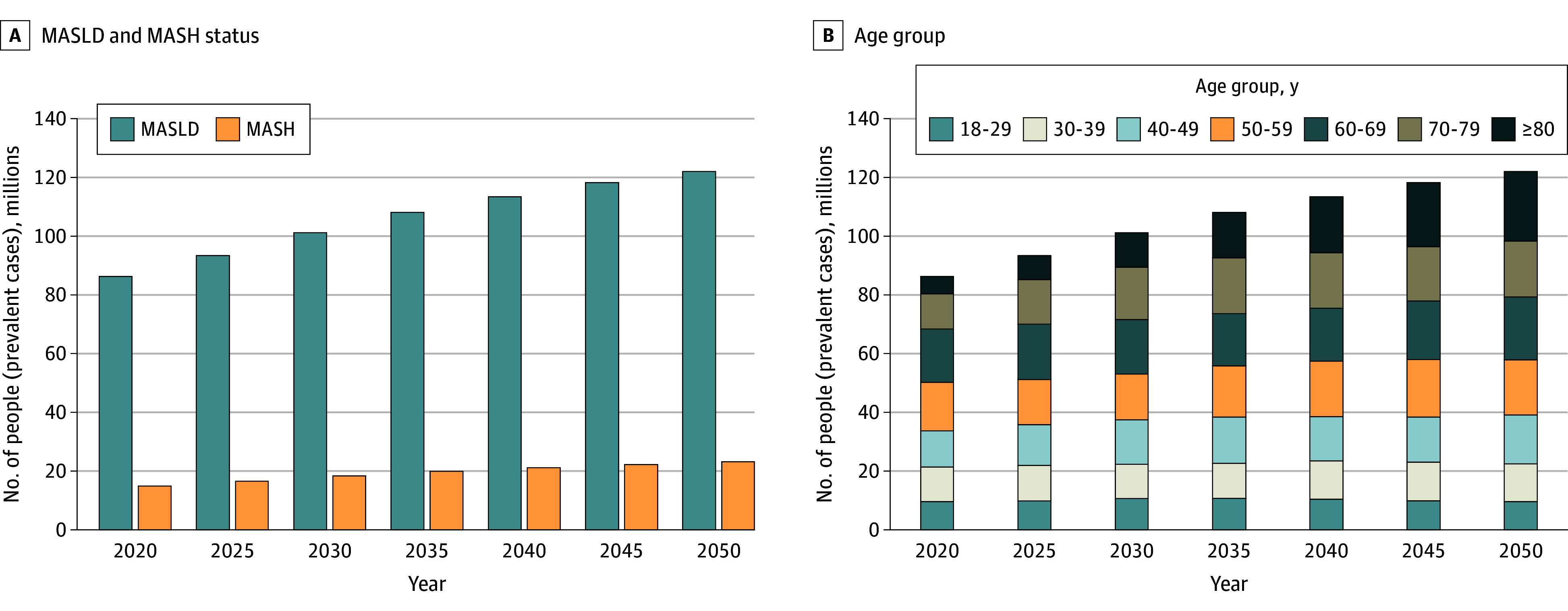
Model Prediction of Metabolic Dysfunction–Associated Steatotic Liver Disease (MASLD) Burden From 2020 to 2050, by MASLD and Metabolic Dysfunction–Associated Steatohepatitis (MASH) Status and by Age Group

**Figure 3.**
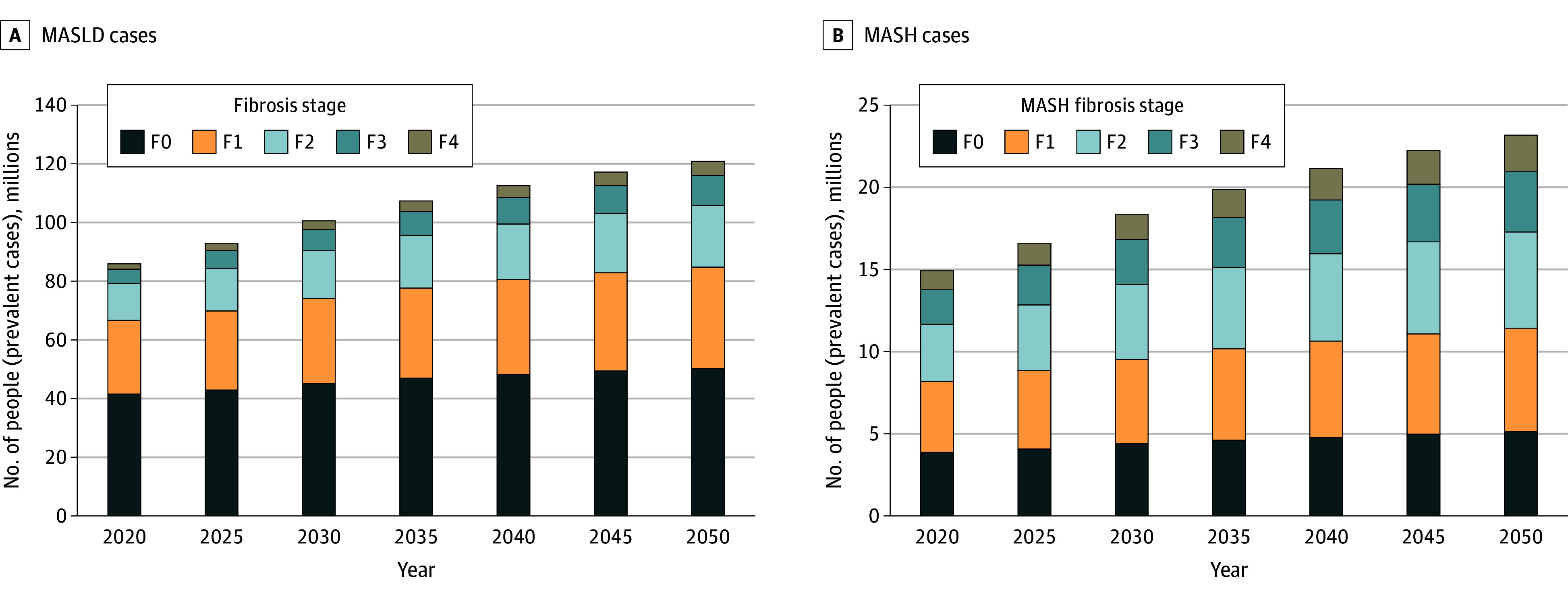
Model Prediction of Metabolic Dysfunction–Associated Steatotic Liver Disease (MASLD) Burden From 2020 to 2050 by Fibrosis Stage for All MASLD and Metabolic Dysfunction–Associated Steatohepatitis (MASH) Cases F0 to F4 represents fibrosis stage 0 (no fibrosis) to 4 (cirrhosis).

In the base-case, the model estimated that 33.7% of the US adult population, or 86.3 million people, had MASLD in 2020. This would increase 36.8% in 2030, equivalent to 101.2 million people, and 41.4% in 2050, equivalent to 121.9 million people (Figure 2). Case counts increased across age groups, with a 300% increase in people 80 years of age or older, followed by 58% in people aged 70 to 79 years. Stratifying by fibrosis stage, almost half (48.4%) of patients with MASLD did not have fibrosis (F0) in 2020, while 29.1% had F1, 14.5% had F2, 5.7% had F3, and 2.2% had F4. By 2050, disease would be more advanced, with larger proportions having F2 to F4 (41.6% with F0, 28.6% with F1, 17.4% with F2, 8.4% with F3, and 4.0% with F4) (Figure 3). Decompensated cirrhosis would increase 3.6 times, from 289 200 cases in 2020 to 1 027 400 cases in 2050.

The model also predicted that 5.8% of US adults, or 14.9 million people, had MASH in 2020, increasing 6.7%, or 18.4 million people, by 2030 and 7.9%, or 23.2 million people, by 2050. The population with MASH and F stage of F2 or higher increased 75%, from 6.7 million in 2020 to 11.7 million in 2050. The prevalence increased across fibrosis stages, with higher rates among more advanced stages. In particular, the number of patients with MASH cirrhosis (F4) increased by 91.1%, from 1.147 million in 2020 to 2.192 million in 2050.

### HCC, LT, and Mortality

**Figure 4.**
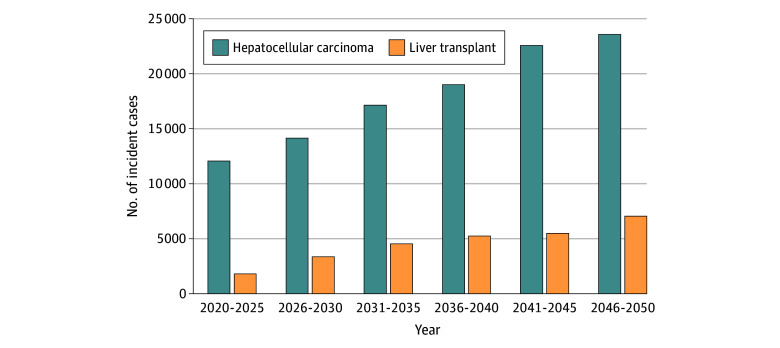
Model Prediction of Annual Incident Metabolic Dysfunction–Associated Steatotic Liver Disease–Related Hepatocellular Carcinoma and Liver Transplant Between 2020 and 2050

The model predicted a mean of 11 483 new cases of HCC and 1717 LTs per year from 2020 through 2025, increasing to 22 440 new cases of HCC and 6720 LTs per year from 2046 through 2050 (Figure 4), for a cumulative total of 527 900 new MASLD-related HCC cases and 132 600 LTs across 30 years. By 2050, the number of HCC cases almost doubled (22 440) and LTs almost quadrupled (6720). Concurrently, MASLD-related liver mortality was 30 500 incident deaths (1.0% of 3 026 300 all-cause deaths among US adults) in 2020, increasing to 95 300 incident deaths (2.4% of 4 052 800 all-cause deaths) in 2050.

### Sensitivity Analysis

When the incidence of MASLD remained stable after 2014, the best-case scenario, its prevalence reached 38.2% or 112.7 million people by 2050, of whom 23.2 million people had MASH (eFigure 7 in Supplement 1). When incidence of MASLD continued to increase after 2030, the worst-case scenario, prevalence rose to 42.9% or 126.5 million people. Under these scenarios, between 21.5 and 23.8 million people had MASH in 2050 (eFigure 7 in Supplement 1). Further analyses are provided in eAppendix 2 and eFigures 8-10 in Supplement 1.

## Discussion

Using an agent-based microsimulation model and assuming that current trends continue, this decision analytical modeling study estimated that the burden of MASLD in US adults would increase markedly by 2050. In the model, the prevalence of MASLD rose to 41.4% (121.9 million adults) by 2050, of whom 23.2 million had MASH. In age-stratified analysis, the fastest increase in prevalence occurred among individuals 80 years of age or older, associated with both age-related incidence and the aging process. More concerning, the model predicted a disproportionate increase in fibrosis stage F2 or higher and related complications, including DC, HCC, and LTs. By 2050, cases of DC were 3.6 times as high, while cases of MASLD-related HCC nearly doubled and LTs almost quadrupled. In sensitivity analyses meant to establish the range of plausible outcomes, between 112.7 and 126.5 million US adults had MASLD by 2050.

Previous studies have projected widely differing burdens of MASLD, which were likely influenced by model assumptions that are not compatible with observed data. Two studies assumed that the annual incidence would remain stable throughout the time horizon of their models.^22,24^ However, given increasing rates of obesity and diabetes^43,44^ and the observed increase in MASLD incidence between 1997 and 2014,^28^ such an assumption leads to underestimates of MASLD, MASH, and their complications. Indeed, their estimates of MASLD and MASH prevalence were one-half or less compared with our estimates.^24^ A third study reported predicted a prevalence of MASH in 2039 that was comparable to our study.^45^ Because the authors used a fixed, high mortality rate for HCC instead of a decreasing rate by years since diagnosis as reported in SEER and a lower mortality rate for LT than UNOS data, they estimated a higher prevalence of LTs and a lower prevalence of HCC cases compared with ours. These studies also offered limited validation. The authors of one study validated their esimate against another model-based prediction,^22^ while the authors of the other studies did not validate their prediction.^24,45^ A fourth study predicted that 33.5% of people aged 15 years or older would have MASLD by 2030, of whom 27% would have MASH.^23^ While their MASLD prevalence was comparable to ours, the estimated MASH prevalence was likely overestimated, although the study did not report burden for people 18 years of age or older separately. They assumed 20% of patients with MASLD had MASH in 2015, their model starting point, which was higher than that estimated from the 2017 to 2018 NHANES data (ie, 17.2%). However, their projection of HCC cases was underestimated because they assumed that fewer than 15% of HCC cases were due to MASH in their back calculations to determine HCC incidence rates. In addition, the authors’ assumption of a constant number of LTs after 2014 contradicted the increasing patterns observed in UNOS data. An accurate estimate of disease burden is important for planning purposes. While an overestimation can lead to misallocation of resources and unnecessary anxiety among the population, underestimation may result in inadequate planning and delayed implementation of preventive measures. By using a microsimulation approach to allow for individual-level disease progression and validating and calibrating it against recent data on the incidence and prevalence of MASLD and its complications, our study provided the most up-to-date and accurate projection of its burden.

Our findings have important implications. While our projected increase in MASLD prevalence poses a significant challenge for health care systems, it is not inevitable. In our model, we assumed that there would be no change in prevention or clinical management strategies for either MASLD or related comorbidities, such as diabetes and obesity. In fact, recent advances in pharmacotherapy for MASLD are promising, with resmetirom therapy leading to MASH resolution and fibrosis improvement in a phase 3 trial.^20,46,47^ The medication was safe and also improved low-density lipoprotein cholesterol, triglyceride, and hepatic fat levels and liver stiffness, and it is the first medication for MASLD approved by the US Food and Drug Administration (FDA).^48^ Other MASLD medication candidates, such as semaglutide, lanifibranor, and pegozafermin, are also in late-stage trials.^49,50,51^ Beyond MASLD, patients prescribed certain FDA-approved diabetes medications have demonstrated remarkable weight loss, comparable to bariatric surgery.^52,53,54^ With more than 60% of patients with MASLD having diabetes, obesity, or both,^55,56^ the uptake of these new drugs may help alter the disease trajectory.

Despite the advent of new treatments, challenges remain. The high cost of new drugs and their rapid price increases could limit patient access,^57^ and long-term adherence with medications presents a challenge.^58^ Identifying patients for treatment, particularly those with fibrotic MASH, and monitoring disease progression will require innovative solutions. Liver biopsy, the current gold standard for diagnosis, has multiple limitations.^59^ Although noninvasive alternatives have been developed and have shown comparable prognostic performance,^60,61,62,63,64^ their utility in monitoring disease progression requires further validation and FDA approval as end points for trials before widespread adoption, creating another barrier to treatment. Studies that adapt our current model to compare screening and treatment strategies could inform future guidelines and identify cost-effective approaches to optimize access.

### Strengths and Limitations

Our model has important strengths. By employing a microsimulation model with state transitions, we provided a comprehensive and granular assessment of the burden of MASLD among US adults, accounting for the dynamic nature of the disease and individual-level variation. While there are no definitive data on the proportion of MASH among patients with MASLD, we are the first to estimate it by using a risk prediction approach and paired liver biopsy data from one of the largest longitudinal cohorts of MASLD patients. We also calibrated unknown model inputs using the most updated prevalence data from NHANES, incidence data from SEER, and transplant data from UNOS. This comprehensive validation using multiple targets and data sources produced well-matched results. Finally, we conducted various sensitivity analyses to assess variation in model estimates under uncertainty.

This study also has limitations. The observational data on which the model is based contain potential inaccuracies, incompleteness, and biases that introduce uncertainty to the projections. The model also contains assumptions regarding disease progression, which may not fully reflect real-world complexities. However, comprehensive calibration and validation were performed to minimize the potential error in model projections. Third, the model does not consider the impact of future interventions and technological advancements on the disease burden. Instead, we focused on the natural progression of the disease to offer valuable insights into the most severe burden. Fourth, the NASH CRN might not be representative of the typical patient with MASH; nevertheless, it is one of the largest registries of MASLD and MASH patients in the US. Finally, because our study is based on specific population data and assumptions, generalizability across diverse populations is limited.

## Conclusions

This decision analytical modeling study estimates a substantial burden of MASLD in the next 30 years in the US. By implementing preventive strategies, investing in research, and preparing health care systems, we can minimize the impact of MASLD and improve the lives of millions of individuals affected by this disease.

## Notes

Supplement 1. **eAppendix 1.** Supplementary Methods **eAppendix 2.** Supplementary Results **eTable 1.** Model Assumptions for the MASLD Natural History Model **eTable 2.** Specifications of the Linear Regression Model for Incidence of Metabolic Dysfunction–Associated Steatotic Liver Disease Among People Aged 18 to 39 years Using Data From Allen AM et al **eTable 3.** Specifications of the Linear Regression Model for Incidence of Metabolic Dysfunction–Associated Steatotic Liver Disease Among People Aged 40 to 59 Years Using Data From Allen AM et al **eTable 4.** Specifications of the Linear Regression Model for Incidence of Metabolic Dysfunction–Associated Steatotic Liver Disease Among People Aged ≥60 Years Using Data From Allen AM et al **eTable 5.** Specifications of the Linear Regression Model for Prevalence of Metabolic Dysfunction–Associated Steatotic Liver Disease Among People Aged ≥18 Years Using NHANES 2001-2018 **eTable 6.** Overall and Age-Specific Prevalence of Metabolic Dysfunction–Associated Steatotic Liver Disease Among People Aged ≥18 Years, Model Prediction vs National Health and Nutrition Examination Survey 2017-2018 **eTable 7.** Multivariable Logistics Model for Predicting MASH Among Patients With MASLD in the NASH CRN Database **eTable 8.** Characteristics of the MASH Prediction Model **eTable 9.** Specifications of the Linear Regression Model for Proportion of Metabolic Dysfunction–Associated Steatohepatitis Among People With Nonalcoholic Fatty Liver Disease Aged ≥18 Years **eTable 10.** US Population Count 2000-2020 By Model Prediction vs Census Bureau Estimate **eFigure 1.** Prevalence of Metabolic Dysfunction–Associated Steatotic Liver Disease (MASLD) Among People Aged ≥18 Years and the Proportion of Metabolic Dysfunction–associated Steatohepatitis (MASH) Among MASLD Patients, National Health and Nutrition Examination Survey 2001-2002 to 2017-2018 **eFigure 2.** Prevalence of MASLD as Predicted by the Model and Estimated From NHANES **eFigure 3.** Proportion of MASH Among MASLD Cases as Predicted by the Model and Estimated From NHANES **eFigure 4.** Cumulative Incidence of Hepatocellular Carcinoma Predicted by the Model Under Different Assumptions on the Proportion of MASH Among MASLD Patients Compared to SEER Database **eFigure 5.** Cumulative Incidence of Liver Transplant Predicted by the Model Under Different Assumptions on the Proportion of MASH Among MASLD Patients Compared to UNOS Database **eFigure 6.** Cumulative Survival of Participants in the NASH CRN Database Compared to Model Prediction **eFigure 7.** Sensitivity Analysis-Model Prediction of MASLD (a) and MASH (b) Cases From 2020 to 2050 in the Base-Case and Worst and Best-Case Scenarios **eFigure 8.** Sensitivity Analysis-Model Prediction of MASLD (a) and MASH (b) Cases From 2020 To 2050 in the Base Case, and Different Assumptions on the Rate of Increase in Prevalence of MASLD Between 2000 and 2018 **eFigure 9.** Sensitivity Analysis-Model Prediction of MASH Cases From 2020 to 2050 in the Base Case With Different Assumptions on the Rate of Increase in Proportion of MASH Between 2000 and 2018 **eFigure 10.** One-Way Sensitivity Analysis-Model Prediction of Incident Hepatocellular Carcinoma Cases Per Year in 2046-2050 by Ranges of Various Model Inputs

Supplement 2.
Data Sharing Statement
