# Supplementary material for: Estimated Burden of Metabolic Dysfunction–Associated Steatotic Liver Disease in US Adults, 2020 to 2050
Source: JAMA Netw Open. 2025 Jan 17;8(1):e2454707. doi: 10.1001/jamanetworkopen.2024.54707 (PMC11742522; doi:10.1001/jamanetworkopen.2024.54707)
Supplement: Supplement 1. — eAppendix 1. Supplementary Methods eAppendix 2. Supplementary Results eTable 1. Model Assumptions for the MASLD Natural History Model eTable 2. Specifications of the Linear Regression Model for Incidence of Metabolic Dysfunction–Associated Steatotic Liver Disease Among People Aged 18 to 39 years Using Data From Allen AM et al eTable 3. Specifications of the Linear Regression Model for Incidence of Metabolic Dysfunction–Associated Steatotic Liver Disease Among People Aged 40 to 59 Years Using Data From Allen AM et al eTable 4. Specifications of the Linear Regression Model for Incidence of Metabolic Dysfunction–Associated Steatotic Liver Disease Among People Aged ≥60 Years Using Data From Allen AM et al eTable 5. Specifications of the Linear Regression Model for Prevalence of Metabolic Dysfunction–Associated Steatotic Liver Disease Among People Aged ≥18 Years Using NHANES 2001-2018 eTable 6. Overall and Age-Specific Prevalence of Metabolic Dysfunction–Associated Steatotic Liver Disease Among People Aged ≥18 Years, Model Prediction vs National Health and Nutrition Examination Survey 2017-2018 eTable 7. Multivariable Logistics Model for Predicting MASH Among Patients With MASLD in the NASH CRN Database eTable 8. Characteristics of the MASH Prediction Model eTable 9. Specifications of the Linear Regression Model for Proportion of Metabolic Dysfunction–Associated Steatohepatitis Among People With Nonalcoholic Fatty Liver Disease Aged ≥18 Years eTable 10. US Population Count 2000-2020 By Model Prediction vs Census Bureau Estimate eFigure 1. Prevalence of Metabolic Dysfunction–Associated Steatotic Liver Disease (MASLD) Among People Aged ≥18 Years and the Proportion of Metabolic Dysfunction–associated Steatohepatitis (MASH) Among MASLD Patients, National Health and Nutrition Examination Survey 2001-2002 to 2017-2018 eFigure 2. Prevalence of MASLD as Predicted by the Model and Estimated From NHANES eFigure 3. Proportion of MASH Among MASLD Cases as Predicted by the Model and [file jamanetwopen-e2454707-s001.pdf]

Supplementary Online Content

Le P, Tatar M, Dasarathy S, et al. Projection of the clinical burden of metabolic dysfunction-associated steatotic liver disease in US adults, 2020-2050. *JAMA Netw Open*. 2024;8(1):e2454707. doi:10.1001/jamanetworkopen.2024.547070

eAppendix 1. Supplementary Methods

eAppendix 2. Supplementary Results

eTable 1. Model Assumptions for the MASLD Natural History Model

eTable 2. Specifications of the Linear Regression Model for Incidence of Metabolic Dysfunction-Associated Steatotic Liver Disease Among People Aged 18 to 39 years Using Data From Allen AM et al

eTable 3. Specifications of the Linear Regression Model for Incidence of Metabolic Dysfunction-Associated Steatotic Liver Disease Among People Aged 40 to 59 Years Using Data From Allen AM et al

eTable 4. Specifications of the Linear Regression Model for Incidence of Metabolic Dysfunction-Associated Steatotic Liver Disease Among People Aged ≥60 Years Using Data From Allen AM et al

eTable 5. Specifications of the Linear Regression Model for Prevalence of Metabolic Dysfunction-Associated Steatotic Liver Disease Among People Aged ≥18 Years Using NHANES 2001-2018

eTable 6. Overall and Age-Specific Prevalence of Metabolic Dysfunction-Associated Steatotic Liver Disease Among People Aged ≥18 Years, Model Prediction vs National Health and Nutrition Examination Survey 2017-2018

eTable 7. Multivariable Logistics Model for Predicting MASH Among Patients With MASLD in the NASH CRN Database

eTable 8. Characteristics of the MASH Prediction Model

eTable 9. Specifications of the Linear Regression Model for Proportion of Metabolic Dysfunction-Associated Steatohepatitis Among People With Nonalcoholic Fatty Liver Disease Aged ≥18 Years

eTable 10. US Population Count 2000-2020 By Model Prediction vs Census Bureau Estimate

eFigure 1. Prevalence of Metabolic Dysfunction-Associated Steatotic Liver Disease (MASLD) Among People Aged ≥18 Years and the Proportion of Metabolic Dysfunction-

© 2025 Le P et al. *JAMA Network Open*.

Associated Steatohepatitis (MASH) Among MASLD Patients, National Health and Nutrition Examination Survey 2001-2002 to 2017-2018

eFigure 2. Prevalence of MASLD as Predicted by the Model and Estimated From NHANES

eFigure 3. Proportion of MASH Among MASLD Cases as Predicted by the Model and Estimated From NHANES

eFigure 4. Cumulative Incidence of Hepatocellular Carcinoma Predicted by the Model Under Different Assumptions on the Proportion of MASH Among MASLD Patients Compared to SEER Database

eFigure 5. Cumulative Incidence of Liver Transplant Predicted by the Model Under Different Assumptions on the Proportion of MASH Among MASLD Patients Compared to UNOS Database

eFigure 6. Cumulative Survival of Participants in the NASH CRN Database Compared to Model Prediction

eFigure 7. Sensitivity Analysis-Model Prediction of MASLD (a) and MASH (b) Cases From 2020 to 2050 in the Base Case, and Worst and Best-Case Scenarios

eFigure 8. Sensitivity Analysis-Model Prediction of MASLD (a) and MASH (b) Cases From 2020 To 2050 in the Base Case, and Different Assumptions on the Rate of Increase in Prevalence of MASLD Between 2000 and 2018

eFigure 9. Sensitivity Analysis-Model Prediction of MASH Cases From 2020 to 2050 in the Base Case With Different Assumptions on the Rate of Increase in Proportion of MASH Between 2000 and 2018

eFigure 10. One-Way Sensitivity Analysis-Model Prediction of Incident Hepatocellular Carcinoma Cases Per Year in 2046-2050 by Ranges of Various Model Inputs

eReferences.

This supplementary material has been provided by the authors to give readers additional information about their work.

© 2025 Le P et al. *JAMA Network Open*.

eAppendix 1. Supplementary Methods

Study design

We developed an agent-based state transition model with a yearly cycle and lifetime time horizon. An agent-based or microsimulation approach was used to capture heterogeneity within the population, account for individual-level variation, and track the impact of that variation on individual outcomes, leading to a more accurate projection within a population.[1] Agent-based modeling effectively captures heterogeneity in disease progression within populations by simulating individual agents with distinct characteristics (e.g. age and sex in our model) and behaviors, allowing for a more nuanced understanding of how these differences influence outcomes.[2, 3] Unlike traditional Markov cohort models, which rely on fixed transition probabilities to represent disease progression across a homogeneous population, microsimulation model allows for transition probabilities that can vary by patient characteristics (e.g. age-dependent incidence of MASLD) or change over time (e.g. risk of death by years of liver cancer diagnosis or years since liver transplant). This flexibility enables an agent-based model to reflect real-world variability in disease trajectories, accounting for factors such as comorbidities as needed, ultimately providing a richer and more accurate depiction of disease dynamics.[2, 3]

Model assumptions

Supplementary Table 1 summarizes all assumptions used for the MASLD natural history model, as well as sources of data and values used for sensitivity/scenario analysis if applicable.

Prevalence of metabolic dysfunction-associated steatotic liver disease (MASLD) and temporal trends

To estimate the prevalence of MASLD in year 2000, the start of our model, we first estimated prevalence in 2018 using 2017-2018 National Health and Nutrition Examination Survey (NHANES).[4] We defined MASLD as having a controlled attenuation parameter (CAP) score of ≥285 dB/m on vibration-controlled transient elastography (VCTE) with one or more metabolic risk factors and excluded pregnant women and people with high alcohol consumption (>140 gram/week in women and >210 gram/week in men). [5, 6] The age-adjusted prevalence of MASLD in 2018 was estimated at 33.4% (95% CI: 30.3-36.6).

Because earlier NHANES cycles lacked CAP score, we calculated annual prevalence of MASLD from 2001-2002 to 2017-2018 following the MASLD definition above using an alternative definition for liver steatosis — US Fatty Liver Index (USFLI) ≥30.[7] To estimate the rate of change in prevalence over time, we ran a linear regression treating prevalence as the dependent variable and survey year as an independent variable. By this method, the prevalence of MASLD was found to increase at a rate of 0.31% per year (95% CI: 0.054-0.569). We then applied this rate of change to the 2018 prevalence to project the prevalence of MASLD backward to the year 2000. This estimate— 27.8% —was used for the base case. In sensitivity analysis, we varied the prevalence of MASLD in year 2000 from 23.2% to 32.4 % which represented the back calculations using the upper and lower bound of the 95% CI of the rate of change. (Supplementary Tables 5 & 6, Supplementary Figure 1).

© 2025 Le P et al. *JAMA Network Open*.

Metabolic dysfunction-associated steatohepatitis (MASH) prediction model

We built a MASH prediction model for patients with MASLD using a subset of data from the non-interventional registry of the non-alcoholic steatohepatitis (NASH) Clinical Research Network (CRN). The NAFLD Databases 1-3 (NCT01030484 and NCT04454463) included adults patients enrolled in the NASH CRN from 2002 to 2022, as well as those in the placebo arms of the PIVENS (NCT00063622) and FLINT (NCT01265498) randomized controlled trials.[8, 9] This subset of the registry included 452 patients aged ≥18 years with biopsy-proven NAFLD who had paired biopsies (with at least one-year interval) and complete data on demographics, metabolic syndrome, physical examination and laboratory test results. In addition, all exams and tests had to be within 6 months of the biopsy.

We constructed the prediction model using data recorded at baseline and assessed its performance using data recorded at follow-up. The outcome of interest was definite MASH. Patient characteristics included age, sex, race, ethnicity, smoking status, BMI, waist circumference, medical history (hypertension, hyperlipidemia, diabetes, coronary artery disease), medication use (antihyperlipidemic, antidiabetic, antihypertensive), and laboratory tests (platelet count, total bilirubin, aspartate aminotransferase (AST), alanine aminotransferase (ALT), alkaline phosphatase, gamma glutamyl transferase (GGT), albumin, HbA1c, international normalized ratio (INR), triglyceride, total cholesterol, HDL, LDL, fasting glucose, fasting insulin, HOMAIR, systolic and diastolic blood pressure). Variables with a p-value of ≤0.2 in univariate analysis were entered in the multivariable logistic model. Variables with a p-value ≤0.1 were retained in the final model. We reported on the area under the receiver operating characteristics (AUROC) curve and identify the cut-off of predicted probability that minimized the difference between sensitivity and specificity.[10] We also reported sensitivity, specificity, positive and negative predictive values of the model using data at baseline and follow-up.

The mean duration between the first and last biopsies was 4.3 (range: 1-15.6) years. Supplementary Table 7 presents the final logistic model. The AUROC was 0.74 and the optimal cut-off value was 0.65, i.e. patients were considered to have MASH if their predicted probability was >0.65 and not otherwise. Supplementary Table 8 includes the characteristics of the prediction model using patient characteristics at baseline and follow-up of the NASH CRN.

Proportion of MASH and temporal trends

We applied the risk prediction model as described above to estimate the predicted probability of having MASH among patients with MASLD in NHANES from 2001-2002 to 2017-2018. We defined MASH as having the predicted probability >0.65 and estimated the proportion of MASH among MASLD patients by survey cycle. MASLD patients were identified using the USFLI throughout the survey years to make it consistent. To estimate the rate of change, we fit a linear regression to the proportions from 2001 to 2018. The proportion of MASH among MASLD patients increased at a rate of 0.24% (95% CI: 0.084-0.34) per year (Supplementary Table 9). We used this estimate for the base case.

© 2025 Le P et al. *JAMA Network Open*.

To estimate the proportion of MASH in 2018, we identified MASLD patients from NHANES 2017-2018 using the CAP score of ≥285 dB/m with one or more cardiometabolic risk factors as described above. [5, 6] Subsequently, we applied the risk prediction model and calculated the proportion of MASH in MASLD patients. We estimated that 17.2% (95% CI: 14.7-19.9) of US adult patients with MASLD had MASH in 2018. Using the estimated rate of change of 0.24% per year, we extrapolated the proportion of MASH in year 2000 to be 12.9%. When we extrapolated using the upper and lower bound of 95% CI of the rate of change, the proportion of MASH in year 2000 ranged from 10.2% to 15.6%. We used these estimates for sensitivity analysis.

Model calibration and validation

We used calibration to determine uncertain parameters including age-specific rates of increase in MASLD incidence using MASLD prevalence trends as targets, rates of development and resolution of MASH using MASH proportion trends as targets, and incidence of LT using annually reported cases of MASLD-related HCC and LT as targets.

We validated model outputs against several targets. AnyLogic software uses the OptQuest (OptTek Inc., Boulder, CO) optimizer to find the optimal parameter values that minimize the difference between observed and predicted outcomes.

We first compared the model's population projection to the reported US population from 2001-2020. We then compared predicted prevalence of MASLD from 2001-2018 against our estimates of MASLD, defined as a USFLI score of >30, using NHANES 2001-2018. We also compared the predicted age-specific prevalence in year 2018 against NHANES 2017-2018 using a CAP score of ≥285 dB/m with ≥ 1 cardiometabolic risk factors to identify MASLD. Third, we compared the proportion of MASH among MASLD patients as predicted for year 2001-2018 versus our estimates from NHANES 2001-2018 as detailed above.

Because hepatocellular carcinoma (HCC) and liver transplant are important outcomes, we validated model predictions using these targets. We obtained the reported annual incidence of HCC cases from SEER program among people ≥18 years, assuming that incidence in year 2020 equaled that in 2019. Based on the literature, we assigned a proportion of HCC cases due to MASLD using two separate estimates. One study reported the proportion of MASLD-related HCC increased from 8.2% in 2002 to 13.5% in 2012.[11] Another study estimated that the proportion increased from 14.1% in 2004 to 19.7% in 2009.[12] Extrapolating these estimates into subsequent years, we calculated that between 17.7% and 32.0% of HCC cases were due to MASLD in year 2020. To determine MASLD-related liver transplant count from the UNOS database, we included all transplants among adults (≥18 years) with cirrhosis due to fatty liver/MASH as recipient primary diagnosis,[11] cryptogenic cirrhosis as recipient primary diagnosis and with diabetes or obesity at the time of listing,[13] or HCC and cirrhosis as recipient primary diagnosis with cirrhosis due to fatty liver/MASH as a primary or secondary diagnosis at the time of listing. Because there was a lack of data on annual transition probability from HCC and DCC to liver transplant, we calibrated the incidence of liver transplant and validated the model-predicted incidence of HCC and LT using these targets.

© 2025 Le P et al. *JAMA Network Open*.

Finally, we validated the model outputs against observed outcomes of one of the largest cohorts of MASLD patients. We simulated 1,773 individuals with characteristics similar as those participating in the NASH CRN database who were longitudinally followed up for a median of 4 (ranges: 2.1 to 7.4) years.[14] We estimated and compared predicted versus observed survival, incidence of HCC and liver-related deaths.

Sensitivity analysis

We conducted one-way analysis varying transition probabilities of MASLD progression (Table 1 in main text).

© 2025 Le P et al. *JAMA Network Open*.

eAppendix 2. Supplementary Results

Model validation and calibration

First, the model accurately replicated the growth of US population from 2000 to 2020 (Supplementary Table 10).

Next, we populated the model using incidence estimates from Allen AM et al.[15] The model failed to predict increases in MASLD prevalence over time and underestimated the prevalence in younger patients compared to NHANES data (Supplementary Table 6). Therefore, we calibrated the age-specific rates of increase in MASLD incidence using the prevalence estimated from NHANES 2001-2018 as targets. With the calibrated values, the final MASLD prevalence predicted by the model closely matched NHANES data (Supplementary Figure 2).

Next, we calibrated the rates of developing and resolving MASH using the base case assumption that the rate of increase in MASH proportion was 0.24% per year, which meant MASH proportion increased from 12.9% in 2000 to 17.2% in 2018. Using these rates, the proportion of MASH predicted by the model, again, matched NHANES data (Supplementary Figure 3). The cumulative incidence of HCC and liver transplant from 2001-2020 were estimated from the model using these calibrated rates and compared to SEER and UNOS data. Results suggested that the model-produced estimates matched closely to observed data (Supplementary Figures 4 and 5).

Finally, after simulating a cohort of MASLD patients with characteristics resembling those among patients enrolled in the NASH CRN database for 8 years,[14] our model's predicted survival curve matched reported data (Supplementary Figure 6). Our model estimated that incidence of HCC was 0.45 per 1000 person-years which was within the 95% CI of the observed rate in the NASH CRN (1.11 per 1000 person-years; 95% CI: 0.38-1.83).

Sensitivity analysis

When we held incidence stable after 2030 but varied the rate of increase in prevalence between 2000-2018 within its 95% CI, the total of MASLD and MASH cases in 2050 ranged from 114-128 million and 21.7-24.2 million, respectively (Supplementary Figure 8). Varying the rate of increase in the MASH proportion from 2000-2018 within its 95% CI and holding baseline MASLD prevalence constant led to an estimated 20.9-25.4 million people with MASH in 30 years (Supplementary Figure 9).

Among the variables we included for one-way sensitivity analysis, transition from F3/MASH F3 to DC had the most impact on the annual incidence of HCC in 2046-2050 (Supplementary Figure 10).

© 2025 Le P et al. *JAMA Network Open*.

eTable 1. Model Assumptions for the MASLD Natural History Model

Assumptions for base-case	Source of data and values used in sensitivity analysis
Incidence of MASLD increased until 2023 after which it stabilized	Literature and calibration<br>Worst-case: incidence of MASLD increased indefinitely into the future<br>Best-case: incidence of MASLD increased until 2014, the last year we have data, after which it stabilized
Rates of development and resolution of MASH were the same across fibrosis stages F0-F2	Calibration<br>Not applicable
Only patients with MASL could regress to normal or no liver steatosis	Literature<br>Not applicable
Incidence of HCC among patients with F3/F4 was the same as that among those with MASH F3/F4	Literature<br>95% confidence interval
Incidence of DC among patients with F4 was the same as that among those with MASH F4	Literature<br>95% confidence interval
Once patients progressed to DC, they could not regress to cirrhosis (F4)	Literature<br>Not applicable
Incidence of liver transplant among patients with DC was the same as that among patients with HCC	Calibration<br>Not applicable
Incidence of HCC among patients with DC was half of that among patients with F4/MASH F4	Assumption<br>95% confidence interval

© 2025 Le P et al. *JAMA Network Open*.

eTable 2. Specifications of the Linear Regression Model for Incidence of Metabolic Dysfunction-Associated Steatotic Liver Disease Among People Aged 18 to 39 years Using Data From Allen AM et al[15]

Regression Statistics	
Multiple R	0.982175
R Square	0.964667
Adjusted R Square	0.962459
Standard Error	8.688189
Observations	18

ANOVA

	df	*SS*	*MS*	*F*	*Significance F*
Regression	1	32974.29	32974.29	436.8346	4.85E-13
Residual	16	1207.754	75.48462		
Total	17	34182.05			

	Coefficients	*Standard Error*	*t Statistics*	*P-value*	*Lower 95% Confidence Limit*	*Upper 95% Confidence Limit*
Intercept	-16446.9	791.6013	-20.7767	5.31E-13	-18125	-14768.8
X Variable 1	8.249751	0.394714	20.90059	4.85E-13	7.412995	9.086507

Note: ANOVA = analysis of variance; df = degrees of freedom; MS = mean square; SS = sum of squares.

© 2025 Le P et al. *JAMA Network Open*.

eTable 3. Specifications of the Linear Regression Model for Incidence of Metabolic Dysfunction-Associated Steatotic Liver Disease Among People Aged 40 to 59 Years Using Data From Allen AM et al[15]

Regression Statistics	
Multiple R	0.970281
R Square	0.941446
Adjusted R Square	0.937786
Standard Error	31.6268
Observations	18

ANOVA

	df	*SS*	*MS*	*F*	*Significance F*
Regression	1	257317.9	257317.9	257.2524	2.79E-11
Residual	16	16004.07	1000.255		
Total	17	273322			

	Coefficients	*Standard Error*	*t Statistics*	*P-value*	*Lower 95% Confidence Limit*	*Upper 95% Confidence Limit*
Intercept	-45971.4	2881.592	-15.9535	3.02E-11	-52080.1	-39862.7
X Variable 1	23.0456	1.43684	16.03909	2.79E-11	19.99964	26.09157

Note: ANOVA = analysis of variance; df = degrees of freedom; MS = mean square; SS = sum of squares.

© 2025 Le P et al. *JAMA Network Open*.

eTable 4. Specifications of the Linear Regression Model for Incidence of Metabolic Dysfunction-Associated Steatotic Liver Disease Among People Aged ≥60 Years Using Data From Allen AM et al[15]

Regression Statistics	
Multiple R	0.954486
R Square	0.911043
Adjusted R Square	0.905484
Standard Error	46.38496
Observations	18

ANOVA

	df	*SS*	*MS*	*F*	*Significance F*
Regression	1	352562	352562	163.8631	8.02E-10
Residual	16	34425.03	2151.565		
Total	17	386987.1			

	Coefficients	*Standard Error*	*t Statistics*	*P-value*	*Lower 95% Confidence Limit*	*Upper 95% Confidence Limit*
Intercept	-53836.4	4226.243	-12.7386	8.62E-10	-62795.6	-44877.2
X Variable 1	26.97559	2.107319	12.8009	8.02E-10	22.50827	31.4429

Note: ANOVA = analysis of variance; df = degrees of freedom; MS = mean square; SS = sum of squares.

© 2025 Le P et al. *JAMA Network Open*.

eTable 5. Specifications of the Linear Regression Model for Prevalence of Metabolic Dysfunction-Associated Steatotic Liver Disease Among People Aged ≥18 Years Using NHANES 2001-2018

Regression Statistics	
Multiple R	0.733513982
R Square	0.538042762
Adjusted R Square	0.47204887
Standard Error	1.689178885
Observations	9

ANOVA

	df	*SS*	*MS*	*F*	*Significance F*
Regression	1	23.26292629	23.26292629	8.152917671	0.024500733
Residual	7	19.97327713	2.853325304		
Total	8	43.23620342			

	Coefficients	*Standard Error*	*t Statistics*	*P-value*	*Lower 95% Confidence Limit*	*Upper 95% Confidence Limit*
Intercept	-594.59	219.11	-2.7137	0.0300	-1112.71	-76.49025
X Variable 1	0.311334	0.1090	2.8553	0.0245	0.05350	0.56916

Note: Prevalence of Metabolic Dysfunction-associated Steatotic Liver Disease was estimated using the National Health and Nutrition Examination Survey data from 2001-2002 to 2017-2018 and the US Fatty Liver Index (see Supplementary Figure 1). ANOVA = analysis of variance; df = degrees of freedom; MS = mean square; SS = sum of squares.

© 2025 Le P et al. *JAMA Network Open*.

eTable 6. Overall and Age-Specific Prevalence of Metabolic Dysfunction-Associated Steatotic Liver Disease Among People Aged ≥18 Years, Model Prediction vs National Health and Nutrition Examination Survey 2017-2018

	NHANES 2017-2018*	Model projection in 2018 based on Allen AM et al[15] before calibration	Model projection in 2018 after calibration§
Overall	33.4 (30.3-36.6)	26.8	33.2
By age group			
18-29 years	19.6 (14.9-25.4)	19.1	21.8
30-39 years	29.3 (26.2-32.5)	19.9	26.9
40-49 years	36.5 (29.4-44.1)	22.1	30.2
50-59 years	39.6 (31.6-48.1)	30.6	38.3
60-69 years	39.3 (32.6-46.4)	36.8	43.6
70-79 years	47.2 (39.3-55.2)	35.7	42.2
≥80 years	34.4 (27.5-42)	27.6	33.5

Note: * Metabolic Dysfunction-associated Steatotic Liver Disease was defined as US Fatty Liver Index ≥30 without excessive alcohol consumption or other causes liver disease.
§ Calibration targets were based on the assumption that the rate of increase in prevalence was 0.31% per year (Supplementary Table 5), which meant the prevalence of MASLD in 2000 was 27.8%.

© 2025 Le P et al. *JAMA Network Open*.

eTable 7. Multivariable Logistics Model for Predicting MASH Among Patients With MASLD in the NASH CRN Database

	Odds ratio	Standard Error	z Statistics	P-value	Lower 95% Confidence Limit	Upper 95% Confidence Limit
AST	1.02	0.00	5.17	0.00	1.02	1.03
Alkaline phosphatase	1.01	0.00	1.99	0.05	1.00	1.02
HDL	0.98	0.01	-1.61	0.11	0.97	1.00
Diabetes (yes vs. no	2.85	0.66	4.52	0.00	1.81	4.48

© 2025 Le P et al. *JAMA Network Open*.

eTable 8. Characteristics of the MASH Prediction Model

	Baseline	Follow-up
Area under the receiver operating curve	0.739	NA
Optimal cut-off value for predicted probability	0.650	NA
Sensitivity	0.662	0.596
Specificity	0.647	0.692
Positive predictive value	0.791	0.727
Negative predictive value	0.487	0.555

Note: The MASH prediction model was built using the NASH CRN data (see Supplementary Table 7). Characteristics of the model was calculated with patient characteristics at baseline and at follow-up. NA, not applicable

eTable 9. Specifications of the Linear Regression Model for Proportion of Metabolic Dysfunction-Associated Steatohepatitis Among People With Nonalcoholic Fatty Liver Disease Aged ≥18 Years

Regression Statistics	
Multiple R	0.813412
R Square	0.661639
Adjusted R Square	0.613302
Standard Error	0.984678
Observations	9

ANOVA

	df	*SS*	*MS*	*F*	*Significance F*
Regression	1	13.27175	13.27175	13.68798	0.007657739
Residual	7	6.78714	0.969591		
Total	8	20.05889			

	Coefficients	*Standard Error*	*t Statistics*	*P-value*	*Lower 95% Confidence Limit*	*Upper 95% Confidence Limit*
Intercept	-456.409	127.7257	-3.57335	0.009056	-758.4319042	-154.385
X Variable 1	0.235157	0.063561	3.699728	0.007658	0.084860128	0.385455

Note: Proportion of Metabolic Dysfunction-associated Steatohepatitis among patients with Metabolic Dysfunction-associated Steatotic Liver Disease was estimated using the National Health and Nutrition Examination Survey data from 2001-2002 to 2017-2018, the US Fatty Liver Index, and the MASH prediction model (see Supplementary Figure 1). ANOVA = analysis of variance; df = degrees of freedom; MS = mean square; SS = sum of squares.

© 2025 Le P et al. *JAMA Network Open*.

eTable 10. US Population Count 2000-2020 By Model Prediction vs Census Bureau Estimate

Year	Census Bureau estimate (x 100 persons)	Model prediction	Difference ($\frac{\text{Prediction}-\text{Census estimate}}{\text{Census estimate}} \times 100$)
2000	2,821,624	2,821,624	0
2001	2,849,690	2,849,380	-0.01%
2002	2,876,252	2,873,772	-0.09%
2003	2,901,079	2,899,138	-0.07%
2004	2,928,053	2,925,789	-0.08%
2005	2,955,166	2,952,298	-0.10%
2006	2,983,799	2,979,649	-0.14%
2007	3,012,312	3,007,029	-0.18%
2008	3,040,940	3,035,662	-0.17%
2009	3,067,715	3,064,781	-0.10%
2010	3,093,217	3,093,378	0.01%
2011	3,115,569	3,118,123	0.08%
2012	3,138,310	3,139,826	0.05%
2013	3,159,937	3,161,055	0.04%
2014	3,183,010	3,182,213	-0.03%
2015	3,206,352	3,203,478	-0.09%
2016	3,294,738	3,224,546	-0.15%
2017	3,325,191	3,244,956	-0.15%
2018	3,355,814	3,264,603	-0.07%
2019	3,386,220	3,283,425	0.03%
2020	3,416,462	3,301,168	-0.43%

© 2025 Le P et al. *JAMA Network Open*.

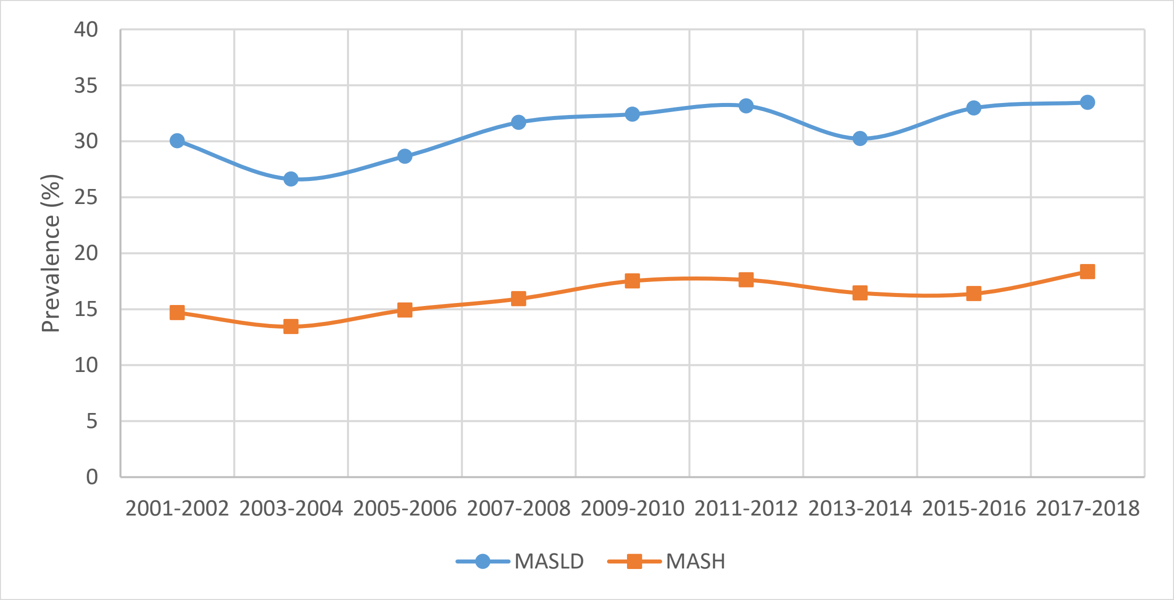

eFigure 1. Prevalence of Metabolic Dysfunction-Associated Steatotic Liver Disease (MASLD) Among People Aged ≥18 Years and the Proportion of Metabolic Dysfunction-Associated Steatohepatitis (MASH) Among MASLD Patients, National Health and Nutrition Examination Survey 2001-2002 to 2017-2018. Metabolic Dysfunction-associated Steatotic Liver Disease was defined as US Fatty Liver Index ≥30 with one or more cardiometabolic risk factors and without excessive alcohol consumption. MASH was defined based on predicted probability of having MASH >0.65. The prediction model was developed using NASH CRN database.

© 2025 Le P et al. *JAMA Network Open*.

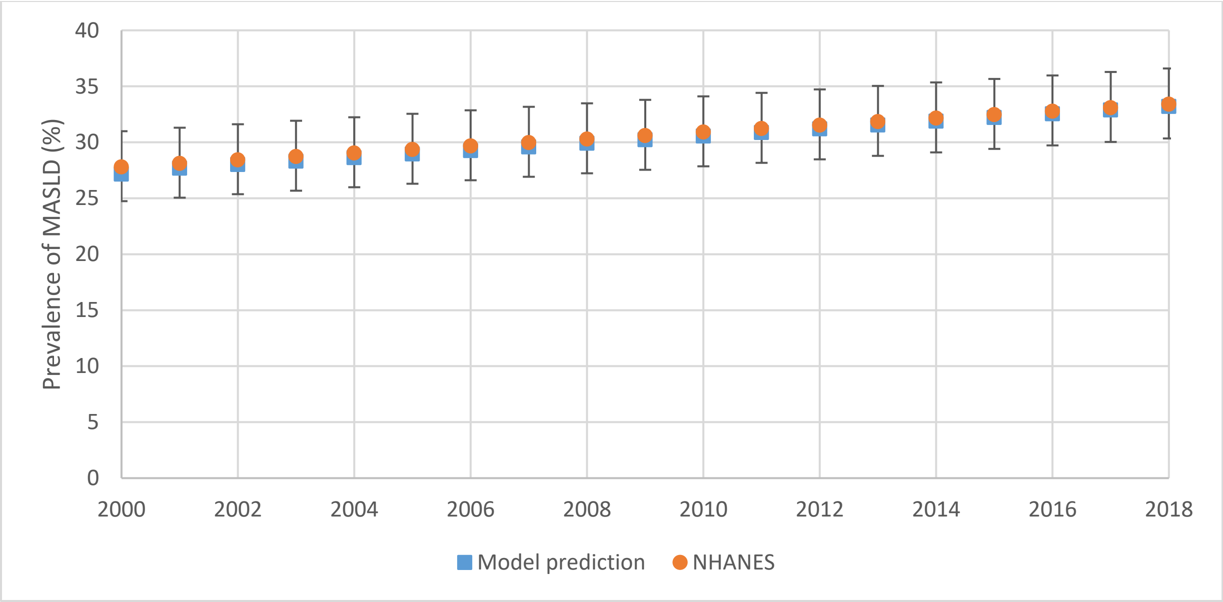

eFigure 2. Prevalence of MASLD as Predicted by the Model and Estimated From NHANES. Error bars represent 95% confidence interval of NHANES estimates. Prevalence of MASLD was estimated from NHANES 2017-2018 using a controlled attenuation parameter score of ≥285 dB/m. The prevalence of MASLD was estimated to be 33.4% in US adults. The prevalence was then retrospectively estimated for earlier years by applying a rate of increase of 0.311% per year (eTable 4).

© 2025 Le P et al. *JAMA Network Open*.

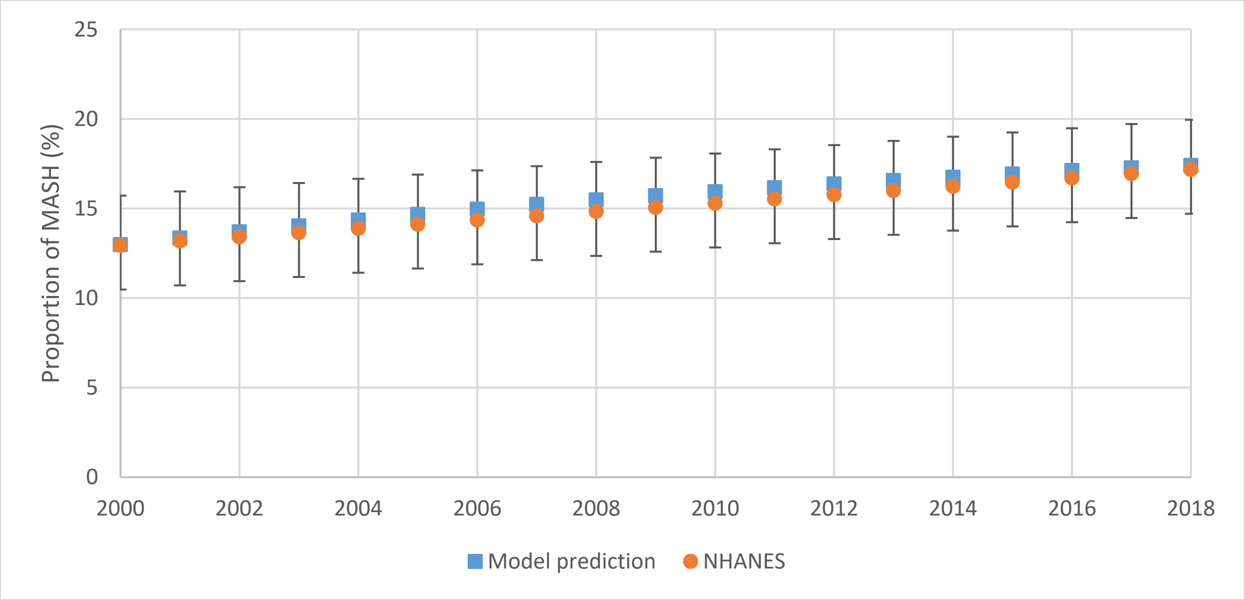

eFigure 3. Proportion of MASH Among MASLD Cases as Predicted by the Model and Estimated From NHANES. Error bars represent 95% confidence interval of NHANES estimates. Proportion of MASH among MASLD was estimated from NHANES 2017-2018 by first using a controlled attenuation parameter score of ≥285 dB/m to define MASLD. The MASH prediction model was then used to identify MASH among people with MASLD (Supplementary Table 6). MASH proportion was estimated to be 17.2% among people with MASLD in NHANES 2017-2018. The proportion of MASH was retrospectively estimated for earlier years by applying a rate of increase of 0.235% per year (eTable 9).

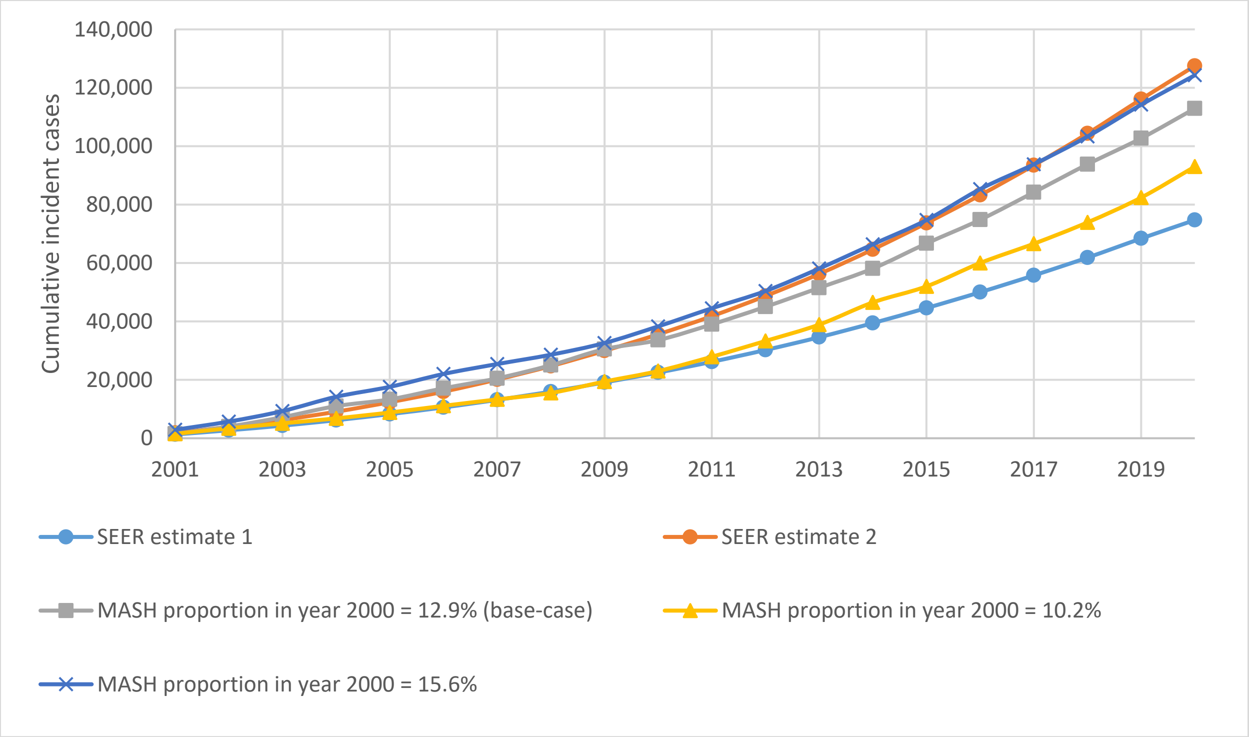

eFigure 4. Cumulative Incidence of Hepatocellular Carcinoma Predicted by the Model Under Different Assumptions on the Proportion of MASH Among MASLD Patients Compared to SEER Database. The two estimates of SEER were based on studies reporting the percentage of MASLD-related hepatocellular carcinoma among all cases.[11, 13]

© 2025 Le P et al. *JAMA Network Open*.

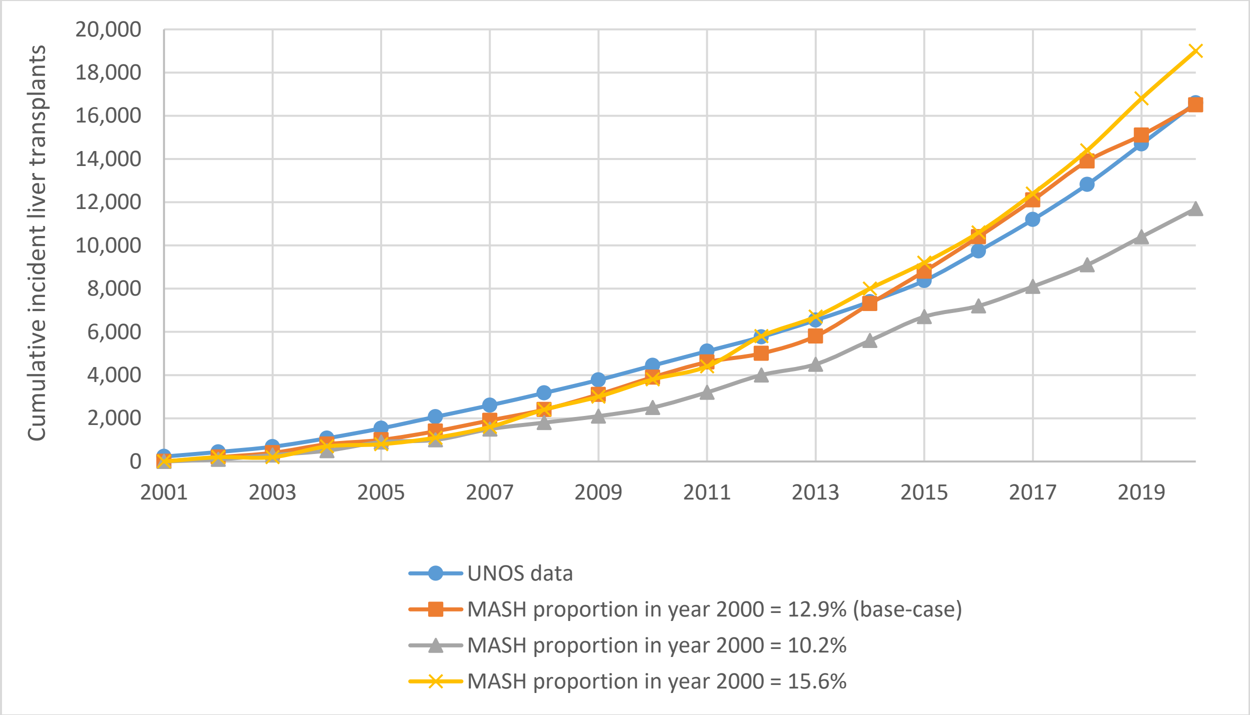

eFigure 5. Cumulative Incidence of Liver Transplant Predicted by the Model Under Different Assumptions on the Proportion of MASH Among MASLD Patients Compared to UNOS Database

© 2025 Le P et al. *JAMA Network Open*.

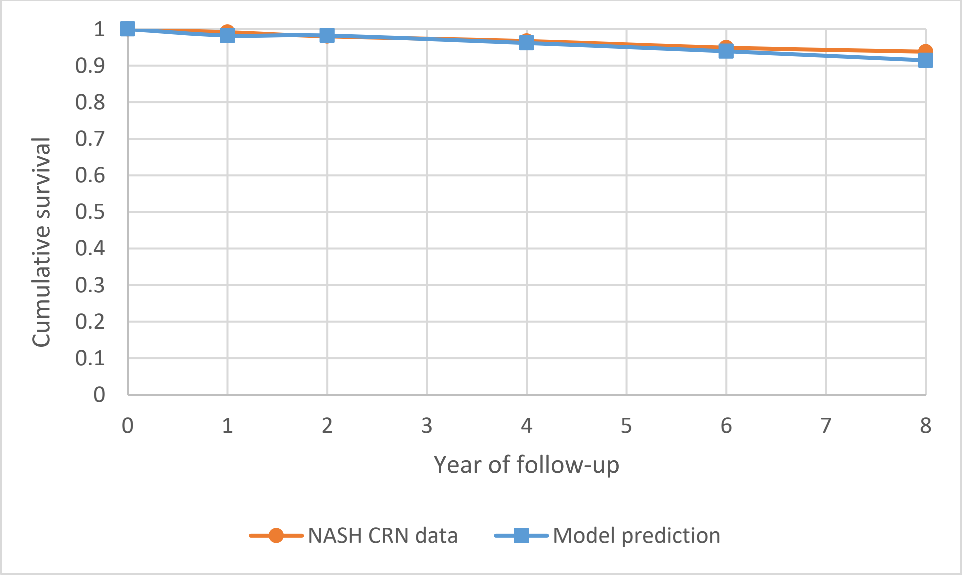

eFigure 6. Cumulative Survival of Participants in the NASH CRN Database Compared to Model Prediction

© 2025 Le P et al. *JAMA Network Open*.

a. MASLD

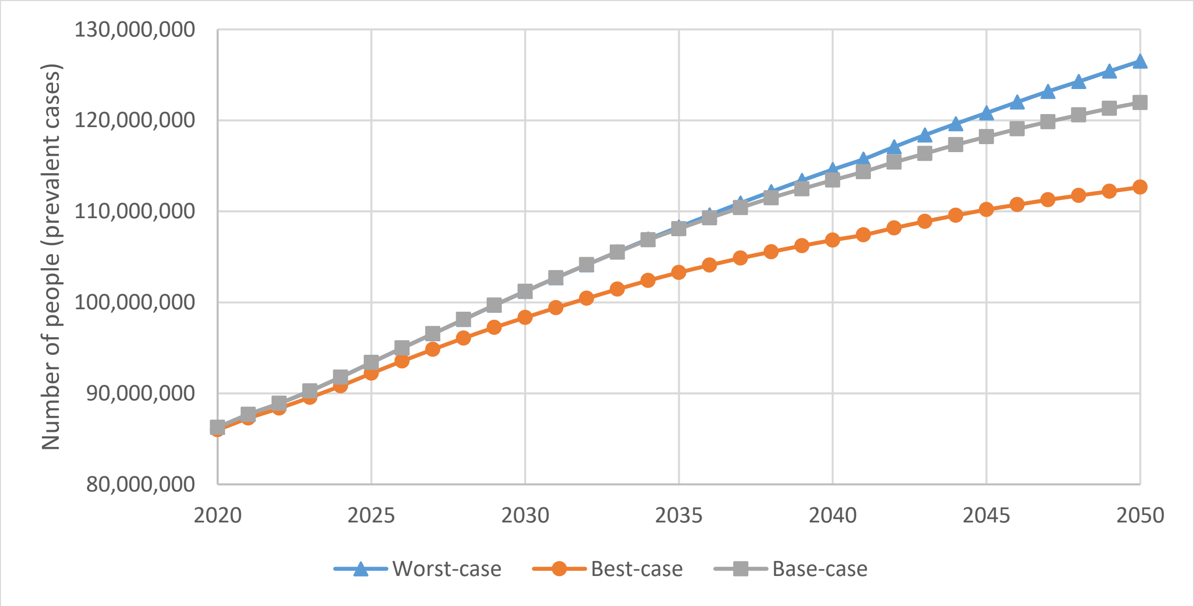

b. MASH

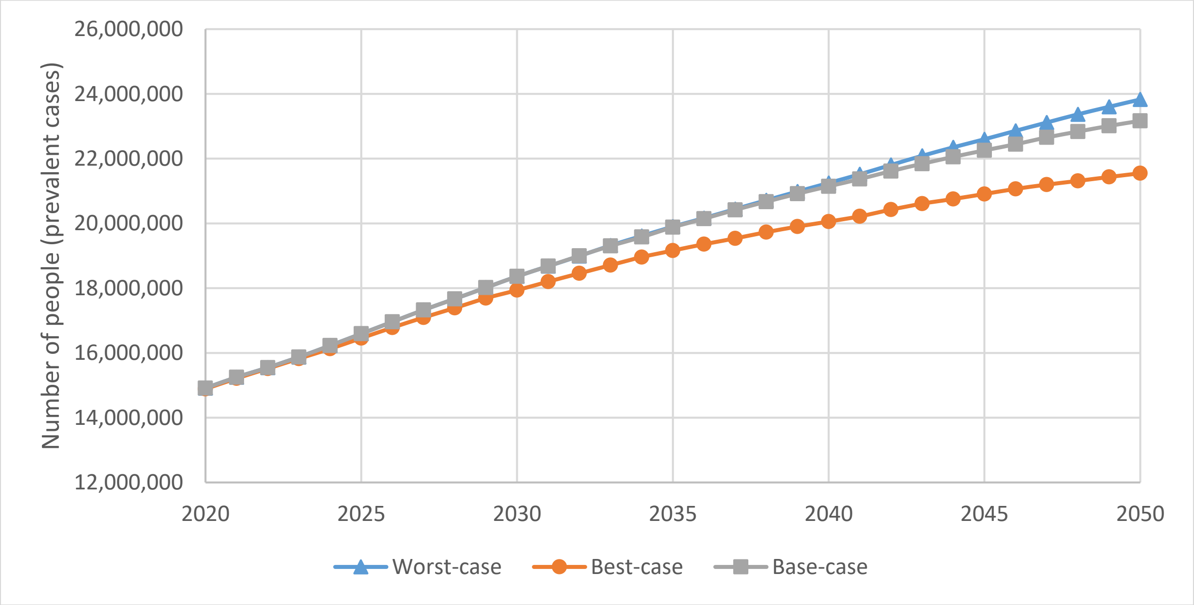

eFigure 7. Sensitivity Analysis-Model Prediction of MASLD (a) and MASH (b) Cases From 2020 to 2050 in the Base Case, and Worst and Best-Case Scenarios. The prevalence of MASLD was assumed to remain stable after 2030 in the base case and after 2014 in the best-case scenario, while increasing after 2030 in the worst-case scenario.

© 2025 Le P et al. *JAMA Network Open*.

a. MASLD

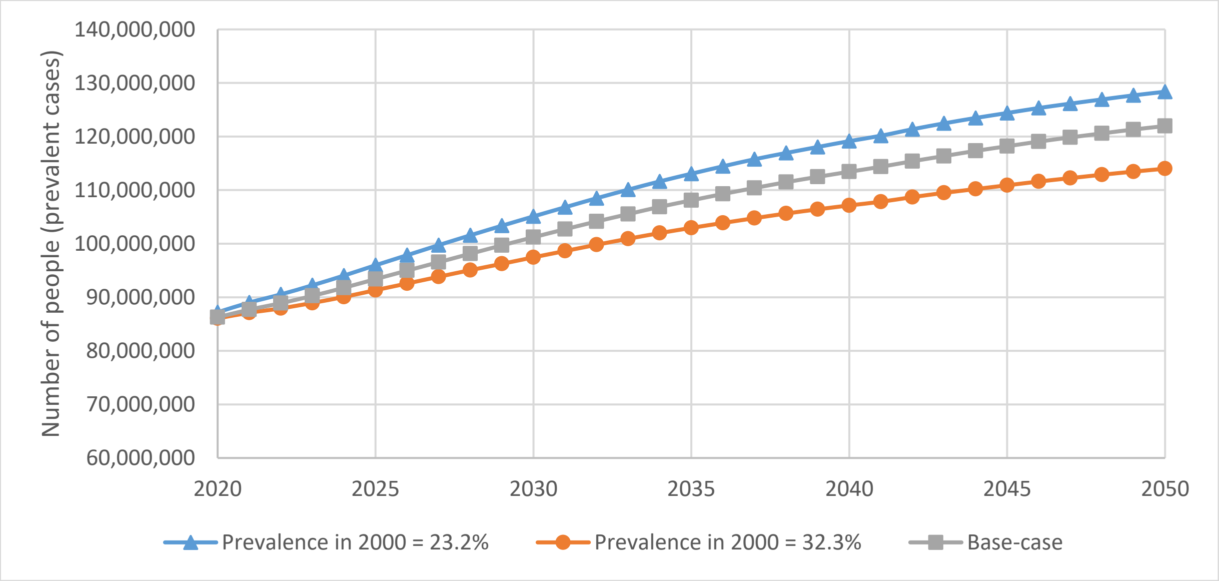

b. MASH

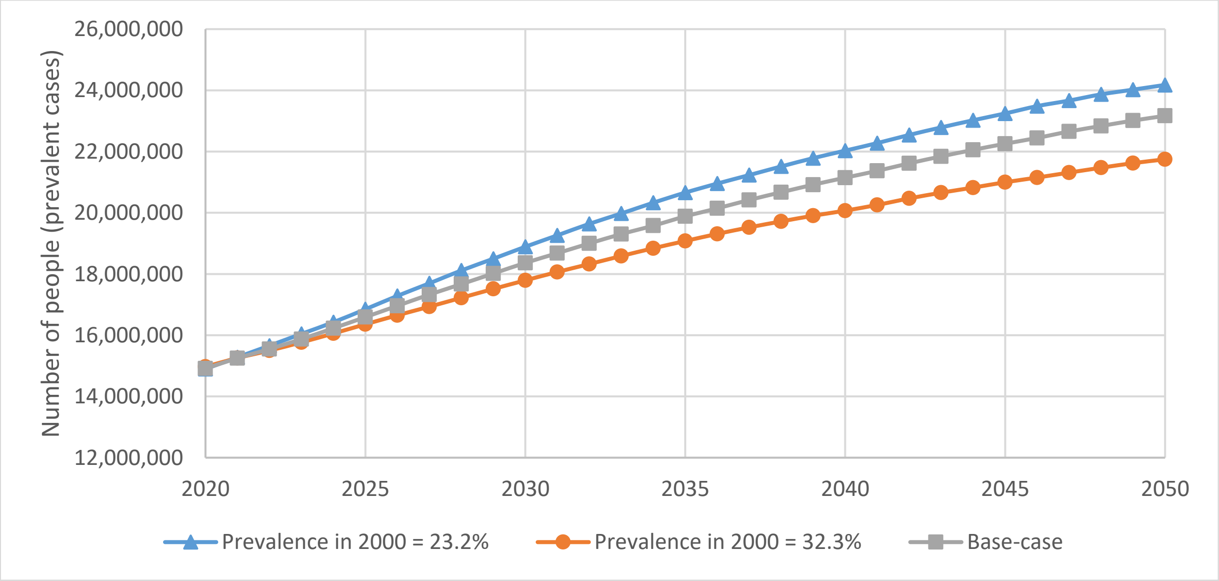

eFigure 8. Sensitivity Analysis-Model Prediction of MASLD (a) and MASH (b) Cases From 2020 To 2050 in the Base Case, and Different Assumptions on the Rate of Increase in Prevalence of MASLD Between 2000 and 2018, i.e., the Lower and Upper Bound of 95% CI of the Rate, Equivalent to a MASLD Prevalence of 32.4% and 23.2% in 2000, Respectively

© 2025 Le P et al. *JAMA Network Open*.

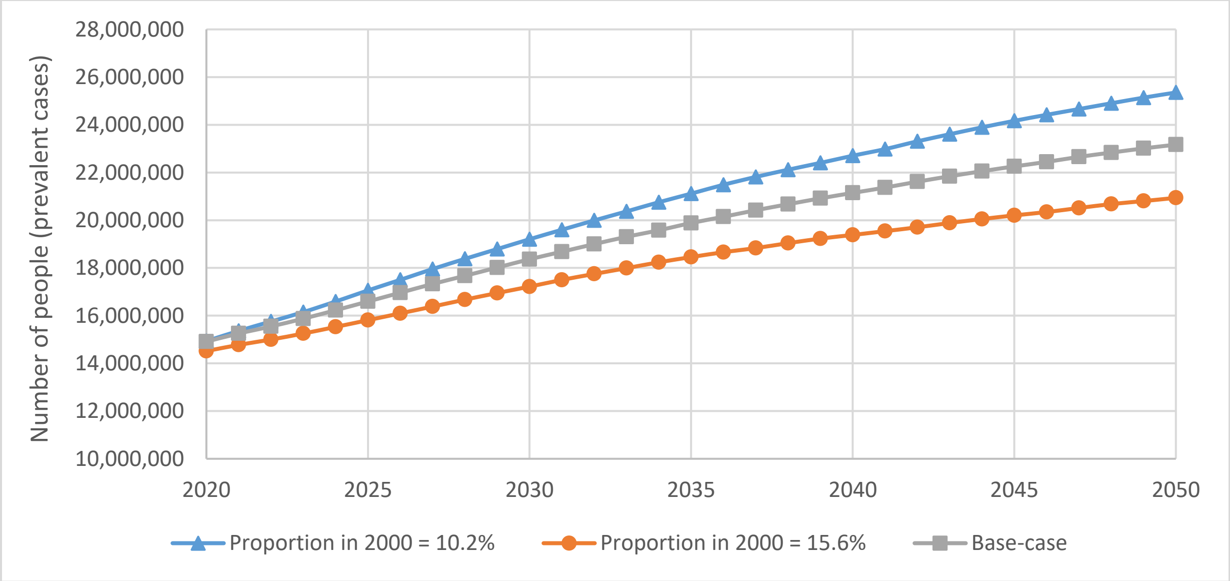

eFigure 9. Sensitivity Analysis-Model Prediction of MASH Cases From 2020 to 2050 in the Base Case With Different Assumptions on the Rate of Increase in Proportion of MASH Between 2000 and 2018, i.e., the Lower and Upper Bound of 95% CI of the Rate, Equivalent to a MASH Proportion of 15.6% and 10.2% in 2000, Respectively

© 2025 Le P et al. *JAMA Network Open*.

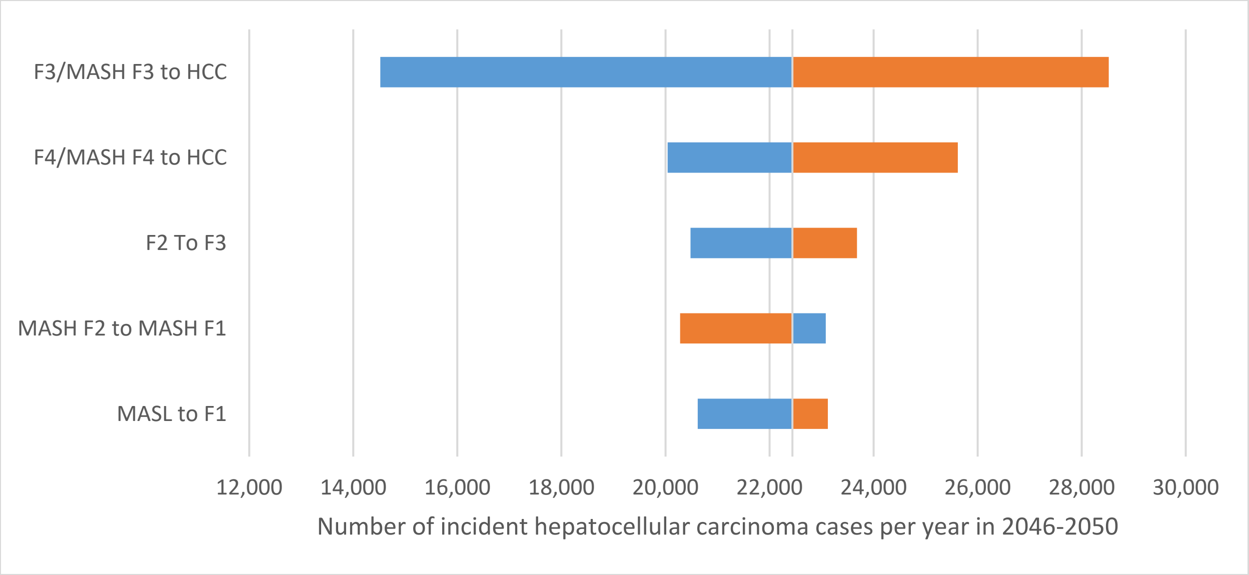

eFigure 10. One-Way Sensitivity Analysis-Model Prediction of Incident Hepatocellular Carcinoma Cases Per Year in 2046-2050 by Ranges of Various Model Inputs

© 2025 Le P et al. *JAMA Network Open*.

eReferences.

1. Marshall DA, Burgos-Liz L, MJ IJ, et al. Selecting a dynamic simulation modeling method for health care delivery research-part 2: report of the ISPOR Dynamic Simulation Modeling Emerging Good Practices Task Force. *Value Health*. Mar 2015;18(2):147-60. doi:10.1016/j.jval.2015.01.006
2. Li Y, Lawley MA, Siscovick DS, Zhang D, Pagan JA. Agent-Based Modeling of Chronic Diseases: A Narrative Review and Future Research Directions. *Prev Chronic Dis*. May 26 2016;13:E69. doi:10.5888/pcd13.150561
3. Tracy M, Cerda M, Keyes KM. Agent-Based Modeling in Public Health: Current Applications and Future Directions. *Annu Rev Public Health*. Apr 1 2018;39:77-94. doi:10.1146/annurev-publhealth-040617-014317
4. Centers for Disease Control and Prevention, National Center for Health Statistics. National Health and Nutrition Examination Survey, 2017-2018. Accessed Jan 30, 2023. https://www.cdc.gov/nchs/nhanes/index.htm
5. Lee BP, Dodge JL, Terrault NA. National prevalence estimates for steatotic liver disease and subclassifications using consensus nomenclature. *Hepatology*. Mar 1 2024;79(3):666-673. doi:10.1097/HEP.0000000000000604
6. Rinella ME, Lazarus JV, Ratziu V, et al. A multisociety Delphi consensus statement on new fatty liver disease nomenclature. *Hepatology*. Dec 1 2023;78(6):1966-1986. doi:10.1097/HEP.0000000000000520
7. Ruhl CE, Everhart JE. Fatty liver indices in the multiethnic United States National Health and Nutrition Examination Survey. *Aliment Pharmacol Ther*. Jan 2015;41(1):65-76. doi:10.1111/apt.13012
8. Neuschwander-Tetri BA, Loomba R, Sanyal AJ, et al. Farnesoid X nuclear receptor ligand obeticholic acid for non-cirrhotic, non-alcoholic steatohepatitis (FLINT): a multicentre, randomised, placebo-controlled trial. *The Lancet*. 2015;385(9972):956-965.
9. Sanyal AJ, Chalasani N, Kowdley KV, et al. Pioglitazone, vitamin E, or placebo for nonalcoholic steatohepatitis. *New England Journal of Medicine*. 2010;362(18):1675-1685.
10. Habibzadeh F, Habibzadeh P, Yadollahie M. On determining the most appropriate test cut-off value: the case of tests with continuous results. *Biochem Med (Zagreb)*. Oct 15 2016;26(3):297-307. doi:10.11613/BM.2016.034
11. Wong RJ, Cheung R, Ahmed A. Nonalcoholic steatohepatitis is the most rapidly growing indication for liver transplantation in patients with hepatocellular carcinoma in the U.S. *Hepatology*. Jun 2014;59(6):2188-95. doi:10.1002/hep.26986
12. Younossi ZM, Otgonsuren M, Henry L, et al. Association of nonalcoholic fatty liver disease (NAFLD) with hepatocellular carcinoma (HCC) in the United States from 2004 to 2009. *Hepatology*. Dec 2015;62(6):1723-30. doi:10.1002/hep.28123
13. Younossi ZM, Stepanova M, Ong J, et al. Nonalcoholic Steatohepatitis Is the Most Rapidly Increasing Indication for Liver Transplantation in the United States. *Clin Gastroenterol Hepatol*. Mar 2021;19(3):580-589 e5. doi:10.1016/j.cgh.2020.05.064
14. Sanyal AJ, Van Natta ML, Clark J, et al. Prospective Study of Outcomes in Adults with Nonalcoholic Fatty Liver Disease. *N Engl J Med*. Oct 21 2021;385(17):1559-1569. doi:10.1056/NEJMoa2029349
15. Allen AM, Therneau TM, Larson JJ, Coward A, Somers VK, Kamath PS. Nonalcoholic fatty liver disease incidence and impact on metabolic burden and death: A 20 year-community study. *Hepatology*. May 2018;67(5):1726-1736. doi:10.1002/hep.29546

© 2025 Le P et al. *JAMA Network Open*.
