# Supplementary material for: Estimated Burden of Metabolic Dysfunction–Associated Steatotic Liver Disease in US Adults, 2020 to 2050
Source: JAMA Netw Open. 2025 Jan 17;8(1):e2454707. doi: 10.1001/jamanetworkopen.2024.54707 (PMC11742522; doi:10.1001/jamanetworkopen.2024.54707)
Supplement: Supplement 2. — Data Sharing Statement [file jamanetwopen-e2454707-s002.pdf]

Data Sharing Statement

Le. Estimated Burden of Metabolic Dysfunction–Associated Steatotic Liver Disease in US Adults, 2020 to 2050. *JAMA Netw Open*. Published January 17, 2025. doi:10.1001/jamanetworkopen.2024.54707

Data
Data available: Yes
Data types: Other (please specify)
Additional Information: Data for model inputs are derived from the published literature which are publicly available.
How to access data: Data for model inputs are derived from the published literature which are publicly available.
When available: With publication

Supporting Documents
Document types: None

Additional Information
Who can access the data: Researchers whose proposed use of the data has been approved by the study group.
Types of analyses: For research purpose
Mechanisms of data availability: With approval from the study group and a signed data access agreement
